# Waking up dormant tumor suppressor genes with zinc fingers, TALEs and the CRISPR/dCas9 system

**DOI:** 10.18632/oncotarget.11142

**Published:** 2016-08-09

**Authors:** Benjamin Garcia-Bloj, Colette Moses, Agustin Sgro, Janice Plani-Lam, Mahira Arooj, Ciara Duffy, Shreyas Thiruvengadam, Anabel Sorolla, Rabab Rashwan, Ricardo L. Mancera, Andrea Leisewitz, Theresa Swift-Scanlan, Alejandro H. Corvalan, Pilar Blancafort

**Affiliations:** ^1^ Cancer Epigenetics group, The Harry Perkins Institute of Medical Research, Perth, WA, 6009, Australia; ^2^ School of Anatomy, Physiology and Human Biology, The University of Western Australia, Perth, WA, 6009, Australia; ^3^ Advanced Center for Chronic Diseases (ACCDiS), Pontificia Universidad Católica de Chile, Santiago, RM, 8330034, Chile; ^4^ Faculty of Medicine, School of Medicine, Pontificia Universidad Catolica de Chile, Santiago, RM, 8330034, Chile; ^5^ School of Biomedical Sciences, Curtin Health Innovation Research Institute, Curtin University, Perth WA 6845, Australia; ^6^ School of Nursing, Virginia Commonwealth University, Richmond, Virginia, 23298, USA

**Keywords:** CRISPR/dCas9, ZF, TALE, tumor suppressor genes, gene reactivation

## Abstract

The aberrant epigenetic silencing of tumor suppressor genes (TSGs) plays a major role during carcinogenesis and regaining these dormant functions by engineering of sequence-specific epigenome editing tools offers a unique opportunity for targeted therapies. However, effectively normalizing the expression and regaining tumor suppressive functions of silenced TSGs by artificial transcription factors (ATFs) still remains a major challenge. Herein we describe novel combinatorial strategies for the potent reactivation of two class II TSGs, *MASPIN* and *REPRIMO*, in cell lines with varying epigenetic states, using the CRISPR/dCas9 associated system linked to a panel of effector domains (VP64, p300, VPR and SAM complex), as well as with protein-based ATFs, Zinc Fingers and TALEs. We found that co-delivery of multiple effector domains using a combination of CRISPR/dCas9 and TALEs or SAM complex maximized activation in highly methylated promoters. In particular, CRISPR/dCas9 VPR with SAM upregulated *MASPIN* mRNA (22,145-fold change) in H157 lung cancer cells, with accompanying re-expression of MASPIN protein, which led to a concomitant inhibition of cell proliferation and induction of apoptotic cell death. Consistently, CRISPR/dCas9 VP64 with SAM upregulated *REPRIMO* (680-fold change), which led to phenotypic reprogramming in AGS gastric cancer cells. Altogether, our results outlined novel sequence-specific, combinatorial epigenome editing approaches to reactivate highly methylated TSGs as a promising therapy for cancer and other diseases.

## INTRODUCTION

The aberrant epigenetic silencing of tumor suppressor genes (TSGs) plays a major role in driving both cancer initiation and tumor evolution. In contrast with class I TSGs, which are frequently associated with loss of function alterations, class II TSGs are solely downregulated by epigenetic mechanisms, such as DNA- and histone-posttranslational modifications, and thus are not altered at the genomic level [1]. The reversible nature of these epigenetic alterations offers unique opportunities for potential therapies. However, gene reactivation of silenced TSGs remains a challenge, as it must overcome the threshold necessary to regain gene function and achieve cancer cell phenotypic reprogramming [2]. Although epigenetic drugs (epidrugs) inhibiting the enzymes that induce and/or maintain this epigenetic state are clinically implemented for the treatment of several cancers, these drugs have revealed a number of off target effects, which may lead to high toxicity and even poor outcomes [3–5]. Accordingly, there is a demand for highly selective activation strategies that are able to upregulate the expression of otherwise dormant genes.

In the recent years the molecular biology field has rapidly evolved to generate novel precision medicine strategies, generally referred as Artificial Transcription Factors (ATFs), for locus-specific manipulation of gene expression. These molecular tools make it possible to target an endogenous locus, reprogram the abnormally silenced epigenetic state and correct gene expression patterns, ultimately treating the disease from within using the cell's innate genomic machinery [6, 7]. ATFs generally consist of a DNA-binding domain (DBD) engineered with an effector domain that modulates the transcriptional activity of a targeted regulatory region, such as a promoter or enhancer [8, 9]. To date, three types of ATFs have been developed: Zinc Fingers (ZFs) [10], Transcription Activator-Like Effectors (TALEs) [11], and the Clustered Regularly Interspaced Short Palindromic Repeats (CRISPR) system associated with the bacterial Cas9 protein [12].

Protein-based ATFs, such as ZFs and TALEs, comprise sequence-specific DBDs directly linked to the effector domain. ZF proteins are one of the most ubiquitous transcription factors found in nature and the first ATF platform developed for (epi)genome engineering [13–15]. However, work done by our group and others [16, 17] has shown that multi-modular ZF proteins have substantial off-target genome-wide effects [16]. To overcome these limitations, alternative modular protein scaffolds, known as TALEs [18, 19], have been developed [20–22]. Even though TALEs are highly specific [23, 24] programmable DNA-binding proteins [25–28], the repetitive nature of their DBD has posed some concerns for their stability *in vivo* when transduced with viral vectors [29, 30].

In contrast to ZFs and TALEs, the recently developed CRISPR system is a programmable, DNA-binding ribonucleoprotein [31, 32]. The platform utilizes the enzyme Cas9, derived from the immune system of bacteria (most commonly *S. pyogenes*), which has helicase activity and two nuclease sites [33]. Cas9 is directed to the target sequence by a chimeric single guide RNA (gRNA), which recognizes the genome by complementary base pairing [34]. Mutation of two key amino acids has generated a catalytically inactive, ‘deactivated’ Cas9 (dCas9), which can be linked to different effector domains for gene expression regulation [35–38]. The simplicity of the design, potential high specificity and multiplexing capacities has positioned the CRISPR/dCas9 system as the current ATF of choice to modulate gene expression patterns. Various epigenetic effector domains have been recently developed to increase the potency of CRISPR/dCas9 in activating targeted genes, such as p300 [39], an H3K9 acetylase core catalytic domain, and VPR [40], a complex made of VP64, p65 and Rta fused in tandem to the C-terminus of dCas9. In addition, a modified gRNA, that incorporates two MS2 RNA aptamers into exposed loops of the scaffold structure of the guide, resulted in the generation of the Synergistic Activation Mediator (SAM) complex, which improved the potency of the transactivation by binding p65 and HSF1 in an activation helper protein [41].

In this study, we explored the potential of CRISPR/dCas9 system in conjunction with protein-based ATFs, ZF proteins and TALEs, to reactivate two dormant class II TSGs, and determined optimized combinatorial strategies able to functionally reactivate tumor suppression in cancer cell models. We focused first on *mammary serine protease inhibitor, MASPIN* [42, 43], as a proof-of-concept TSG for which ZF proteins were generated able to partially reactivate the gene [44–50], in breast and lung malignancies [51–53]. In addition, we validated the capacity of the optimized ATF platforms to reactivate a novel TSG, *tp53 dependent G2 arrest mediator candidate, REPRIMO* [54–56], which has been proposed to have tumor suppressive functions in gastric [57, 58] and thyroid cancers among others [59]. We compared individual ATFs, as well as combinations of multiple platforms and effector domains, to re-activate their expression in several cell lines associated with distinct epigenetic states. We demonstrated the benefit of using multiple activator domains simultaneously to enhance activation in highly silenced cell lines. Moreover, the combinatorial effector domain strategy of the SAM complex proved to be the strongest activator in highly methylated promoter contexts. Our study shows that transiently transfected CRISPR/dCas9 systems were able to induce potent re-activation of dormant TSGs in cancer cell lines, effectively regaining tumor suppressive functions. These findings suggest further applications of epigenetic reprogramming and (re)gain-of-function studies in cancer and other diseases.

## RESULTS

### Systematic comparison of artificial transcription factors for activating a tumor suppressor gene

**Figure 1:**
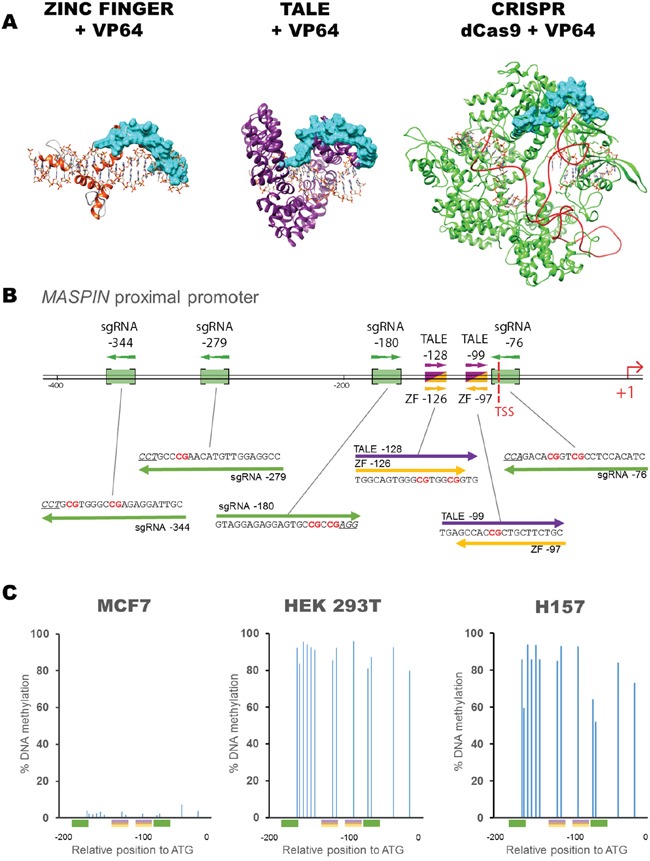
Schematic diagram showing the structure of ATF platforms used, their binding sequences and position within the *MASPIN* proximal promoter **A.** Structural models of ZF (yellow); TALE (purple); and CRISPR/dCas9 (green) with sgRNA (red). All platforms are shown fused to a VP64 effector domain (blue). **B.** Schematic representation of *MASPIN* proximal promoter illustrating ZF (yellow), TALE (purple) and sgRNA (green) binding sites. ZFs and TALEs share overlapping target regions. Protospacer adjacent motif (PAM) sequences of sgRNA guides are underlined and CpG dinucleotides are illustrated in red. TSS refers to transcription start site. **C.** Methylation status of the 13 CpG dinucleotides in the *MASPIN* promoter, within 200 bp upstream of the transcription start site, in un-transfected MCF7 (left), HEK293T (middle) and H157 (right) cells. Colored boxes indicate the various binding sites of ATFs with corresponding sequences for sgRNA-180, TALE-128, ZF-126, TALE-99, ZF-97 and sgRNA-76 (left to right).

We assessed the capacity of three different molecular platforms, previously employed for genome and epigenome editing, to reactivate silenced genes. ZFs, TALEs and CRISPR/dCas9 were each C-terminally linked with the transcriptional activator VP64 (Figure 1A). We chose the silenced tumor suppressor *mammary serine protease inhibitor* (*MASPIN*) as a paradigm of an epigenetically silenced gene, since we had previously shown this gene can be partially reactivated in cell lines bearing high levels of *MASPIN* DNA methylation by 6ZF proteins targeting the proximal promoter: ZF-97 and ZF-126 [44–50]. To compare protein-based ATF backbones, we designed and constructed TALEs targeting 20 bp perfectly overlapping the 18 bp bound by the ZFs with proven upregulation activity. Further, we generated four sgRNAs for CRISPR/dCas9 targeting the promoter region encompassing the ZF/TALE binding sites. The relative positions of all ATF binding sites within the promoter are shown in Figure 1B along with their corresponding target binding sequences (Supplementary Table S1).

We investigated the potential of these molecular tools in three different cell lines, MCF7, HEK293T and H157, all harboring silenced MASPIN protein expression, but with varying levels of *MASPIN* mRNA expression. MCF7 cells had the highest *MASPIN* basal expression, approximately 14 times higher than HEK293T cells and 258 times higher than H157 cells (Supplementary Figure S1). Consistent with their degree of gene silencing, MCF7 cells had low CpG methylation frequency (2.9 ± 1.5% on average), whereas CpG dinucleotides were highly methylated in both HEK293T and H157 cells (89.4 ± 5.4% and 80.5 ± 14% on average, respectively, Figure 1C). The protein expression of each construct in the cells was similar across molecular platforms as assessed by flow cytometry by quantification of green fluorescent protein GFP C-terminally linked to each backbone, which ruled out differences in regulatory capacity due to differential construct expression in the cells (Supplementary Figure S2).

**Figure 2:**
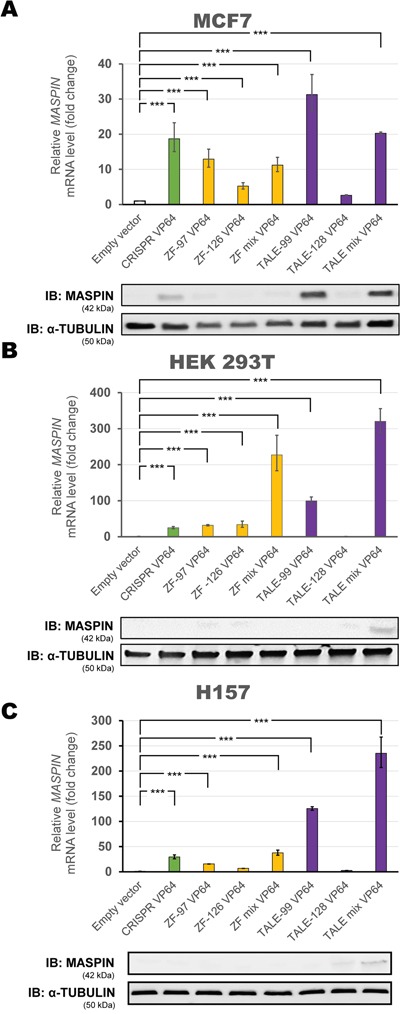
Transient transfection of individual ATFs up-regulates *MASPIN* expression Each platform was transfected in **A.** MCF7, **B.** HEK293T and **C.** H157 cell lines. Top panels: qRT-PCR showing *MASPIN* expression after transfection with various individual ATF platforms: CRISPR/dCas9 VP64 construct with a mix of four gRNAs (green bars), ZF VP64 constructs (yellow bars), and TALE VP64 constructs (purple bars). ZF mix and TALE mix represent an equimolar mix of both ZFs or TALEs, respectively. All qRT-PCR data indicate the fold change in *MASPIN* transcription levels relative to cells transfected with empty vector (pcDNA3.1 backbone), normalized against *GAPDH* expression (error bars represent ± SEM, n=3). Statistical significance: one-way ANOVA, corrected for multiple comparisons using the Tukey HSD test, p<0.05 (*), p<0.01 (**) and p<0.001 (***). Bottom panels: Translation levels of MASPIN (42 kDa) and α-TUBULIN (50 kDa) as shown by a representative immunoblotting (n=2) for each cell line.

In a poorly methylated promoter context with moderate basal *MASPIN* expression (MCF7 cells) individual ATFs significantly increased mRNA expression at different levels, but not all were associated with protein re-expression as assessed by immunoblotting (Figure 2A). TALE-99 VP64 alone or in combination with TALE-128 VP64 were the strongest activators (31.28-fold and 20.3-fold relative to control, respectively, p<0.0001). Consistently, we observed the highest protein expression in these conditions. In contrast, TALE-128 VP64 did not produce significant *MASPIN* upregulation (2.62-fold relative to control, p>0.05). While CRISPR/dCas9 VP64 and ZF VP64 proteins (ZF-97, ZF-126 or a combination of both) induced a significant *MASPIN* mRNA upregulation (18.71-fold compared to 12.92-fold, 5.23-fold and 11.20-fold relative to control, respectively, p<0.0001), only CRISPR/dCas9 VP64 was able to induce protein re-expression.

In the context of a highly methylated promoter with silenced gene expression, such as in the HEK293T (Figure 2B) and H157 cell lines (Figure 2C), we observed that all ATFs individually, except TALE-128, significantly up-regulated *MASPIN* (p<0.0001) at the mRNA level, but failed to translate into significant protein re-expression by immunoblotting. Like in MCF7 cells, TALE-99 was the strongest single activator (99.65-fold and 125.5-fold relative to control in HEK293T and H157 cells, respectively). The combination of both TALEs improved activation even further (320.26-fold and 236.2-fold relative to control in HEK293T and H157 cells, respectively), even though TALE-128 alone did not produce any significant change in expression (0.81-fold and 2.7-fold relative to control in HEK293T and H157, respectively, p>0.05). Similarly, a combination of both ZFs significantly increased *MASPIN* activation compared to ZF-97 and ZF-126 when transfected separately (227.14-fold compared to 31.86- and 33.70-fold relative to control in HEK293T cells, respectively, and 37.5-fold compared to 15.6-fold and 6.8-fold relative to control in H157 cells, respectively). Moreover, delivery of CRISPR/dCas9 VP64 resulted only in moderate transcriptional upregulation (24.95-fold and 29.4-fold relative to control for HEK293T and H157, respectively, p<0.0001). Here again, individual ATFs platforms failed to translate into significant MASPIN expression by immunoblotting in both cell lines.

As expected, the delivery of either dCas9 without an activator domain (CRISPR/dCas9 No Effector), or CRISPR/dCas9 VP64 without any guide targeting *MASPIN* (Supplementary Figure S3), did not produce significant changes in *MASPIN* mRNA expression in HEK293T cells (p>0.05 across all conditions relative to control). This confirmed that CRISPR/dCas9 required sgRNAs to direct the effector domain to the desired target, and that the helicase/topoisomerase activity of dCas9 alone was not able to activate transcription in the absence of a transactivator, such as VP64. These results indicated that ATFs targeted on the same locus had different activating potential depending on the epigenetic background of the cell line. While ATFs in low methylated promoters reactivated tumor suppressor protein expression, in a highly methylated promoter with lower basal expression, they resulted in transcript upregulation yet this was insufficient to elicit protein expression.

In order to improve activation potential, we exploited novel activator domains designed to enhance the effectiveness of the CRISPR/dCas9 system observed by using VP64 alone. To this end, we employed more recently characterized activator effector domains in fusion with the CRISPR/dCas9, including the catalytic histone acetyltransferase core domain of the human E1A-associated protein p300 (CRISPR/dCas9 p300), an epigenetic modifying enzyme, and linked to dCas9 to increase H3K27 acetylation, an epigenetic mark associated with higher transcription activity [39]. In addition, we utilized a strong transactivator complex, VPR (CRISPR/dCas9 VPR), composed of three effectors: VP64, p65 and Rta, linked in tandem to the C-terminus of dCas9 [40]. This construct combined the effect of multiple recruiters of the transcriptional machinery into one single complex and demonstrated a synergic cooperation which significantly improved VP64′s gene activation [40].

**Figure 3:**
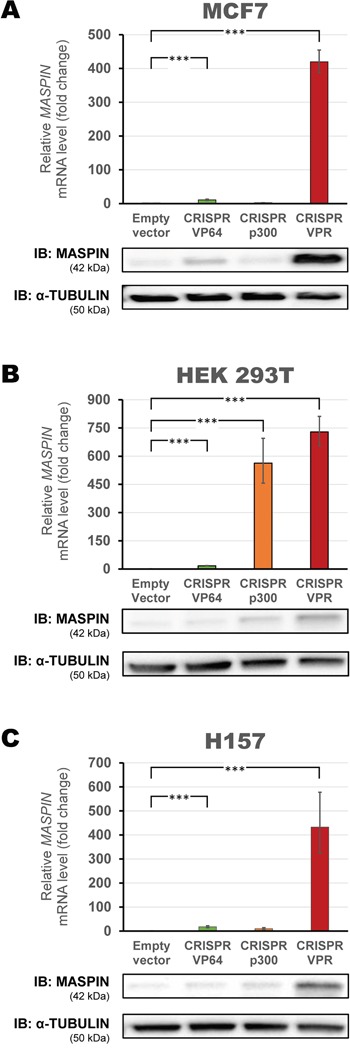
Up-regulation of *MASPIN* expression by second-generation CRISPR/dCas9 p300 and VPR A mix of four gRNAs with dCas9 VP64, dCas9 p300 or dCas9 VPR were transfected into **A.** MCF7, **B.** HEK293T and **C.** H157. Top panels: qRT-PCR data show fold change in *MASPIN* expression for CRISPR/dCas9 VP64 (green), CRISPR/dCas9 p300 (orange) and CRISPR/dCas9 VPR (red), relative to cells transfected with empty vector (pcDNA3.1 backbone), normalized against *GAPDH* expression (error bars represent ± SEM, n=3). Statistical significance: one-way ANOVA, corrected for multiple comparisons using the Tukey HSD test, p<0.001 (***). Translation levels of MASPIN (42 kDa) and α-TUBULIN (50 kDa) as shown by a representative immunoblotting (n=2) are shown for each cell line.

These CRISPR/dCas9 constructs were transiently transfected in the MCF7, HEK293T and H157 cells and their activation potential investigated as described above (Figure 3A–3C). CRISPR/dCas9 VPR greatly increased *MASPIN* mRNA expression in all cell lines (419.8-fold, 728.75-fold and 433.0-fold relative to control in the MCF7, HEK293T and H157 cell lines, respectively, p<0.0001). In contrast, CRISPR/dCas9 p300 did not significantly upregulate *MASPIN* at either mRNA or protein level in MCF7 nor in H157 cells (2.5-fold and 10.2-fold relative to control, respectively, p>0.05). Interestingly, CRISPR/dCas9 p300 was able to significantly upregulate gene expression in HEK293T cells (563.36-fold relative to control, p<0.0001). Protein expression was consistent with qRT-PCR data, and CRISPR/dCas9 VPR elicited the strongest protein upregulation in all cell lines. Overall, CRISPR/dCas9 VPR significantly upregulated *MASPIN* with higher potency than the other activator domains tested. In addition, this construct elicited detectable MASPIN protein re-expression, albeit at higher levels in poorly methylated promoter contexts.

### Combinatorial approach to maximize upregulation of silenced tumor suppressor genes

We next set out to investigate if we could further improve activation efficiency in highly methylated contexts with different ATF tools and effector domains by combining the CRISPR/dCas9 system with protein-based ATFs. We reasoned that the helicase/topoisomerase activity of dCas9 could facilitate chromatin accessibility and act synergistically with protein-based DBDs (ZFs and TALEs) in the context of hyper-methylated promoters to enhance transcriptional upregulation.

**Figure 4:**
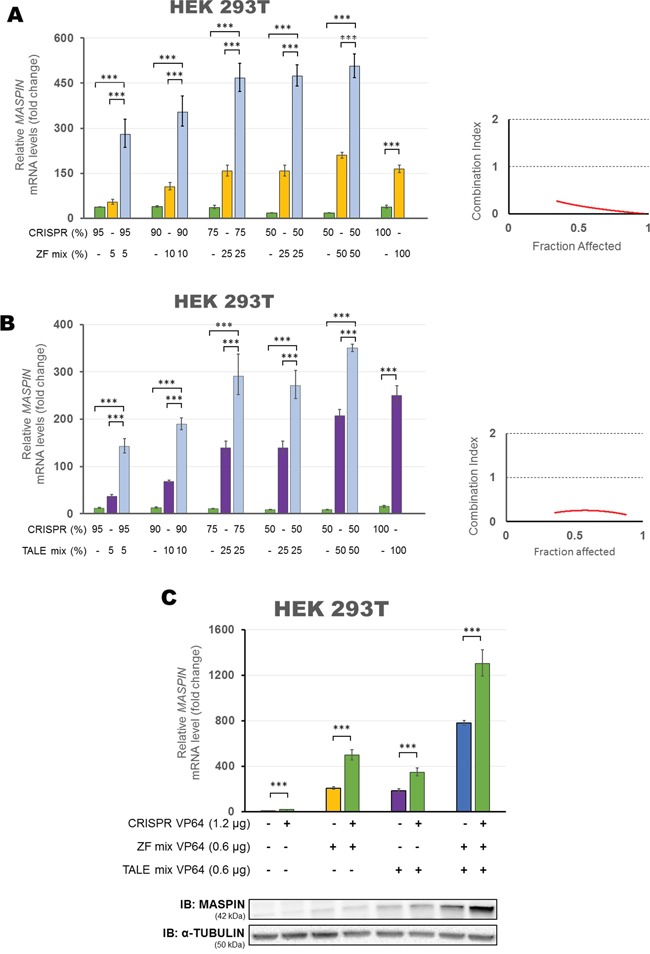
Synergistic up-regulation of *MASPIN* expression by combinations of protein-based ATFs and CRISPR/dCas9 VP64 in HEK293T cells The Combination Index (CI) is plotted for CRISPR/dCas9 VP64 co-transfected with ZF mix **A.** or TALE mix **B.** considering the fold change of mRNA reactivation levels as fraction affected. Left panel: qRT-PCR showing *MASPIN* expression after transfection with varying ratios of CRISPR/dCas9 VP64 (with a mix of four gRNAs) and both ZFs or both TALEs, expressed as a percentage of the total plasmid DNA transfected. Right panel: Combination Index (CI) at each fraction affected, calculated according to the Chou and Talalay method. **C.** Up-regulation of *MASPIN* expression by a triple combination of ATF platforms. HEK293T cells were transfected with a mix of both ZFs VP64 and/or a mix of both TALEs VP64 and/or CRISPR/dCas9 VP64 (with four gRNAs). The platforms delivered in each condition are indicated in the figure at a ratio of 1:1:2, using pcDNA3.1 empty vector to complete total amount of plasmid DNA transfected. Top panel: qRT-PCR data showing *MASPIN* expression. Bottom panel: Translation levels of MASPIN (42 kDa) and α-TUBULIN (50 kDa) as shown by immunoblotting (n=2). All qRT-PCR data indicate the fold change in *MASPIN* transcription levels relative to cells transfected with empty vector, normalized against *GAPDH* expression (error bars represent ± SEM, n=3). Statistical significance: one-way ANOVA, corrected for multiple comparisons using the Tukey HSD test (***, p<0.0001).

We began with the combination of CRISPR/dCas9 VP64 with both ZFs VP64 to determine if there was a pharmacological synergy between these ATFs in upregulating target gene expression (Figure 4A). To assess synergisms, we transfected different ratios of plasmid DNA into HEK293T cells, gradually decreasing the dose of CRISPR/dCas9 from 100% to 50% of the total amount of plasmid DNA transfected per condition, while simultaneously increasing the dose of both ZFs from 0% to 50% of the total amount of plasmid DNA transfected. As expected, we observed a dose-dependent increase in *MASPIN* mRNA levels when both ATFs were transfected separately. However, when transfected in combination, a strong synergistic effect in *MASPIN* upregulation was observed (up to 506.13-fold relative to control when combining 50% CRISPR/dCas9 and 50% ZFs). To determine the interaction between the CRISPR/dCas9 and ZF constructs in reactivating TSG expression we used the Chou and Talalay method [60]. This method calculates a Combination Index (CI) based on the effect of a combination between both agents (CI < 1 being synergistic, CI > 1 antagonistic and CI = 1 additive). In these experiments we considered the maximum fold change reached in *MASPIN* mRNA expression as complete fraction affected, as previously described [46]. We found that the combination of both, ZFs and CRISPR/dCas9 was strongly synergistic (CI<0.3) and similarly, synergistic interactions were observed when each ZF was transfected with CRISPR/dCas9 as single agent (Supplementary Figure S4A).

We next determined pharmacological interactions between TALEs and CRISPR/dCas9 in enhancing gene expression (Figure 4B). When increasing amounts of TALEs and decreasing amounts of CRISPR/dCas9 were transfected, we observed a similar pattern in the dose response to that reported with the ZFs. *MASPIN* activation increased when higher doses of individual ATFs were transfected. More interestingly, a strong synergistic effect in *MASPIN* upregulation was observed again using CRISPR/dCas9 VP64 in combination with TALEs VP64 (up to 350.26-fold relative to control, when combining 50% CRISPR/dCas9 and 50% TALEs). Consequently, we found that TALEs and CRISPR/dCas9 were also strongly synergistic across all doses tested (CI<0.3). However, when using TALEs as single agents, only TALE-99 improved CRISPR/dCas9 activation of *MASPIN* mRNA upregulation (199.3-fold relative to control, p<0.0001, Supplementary Figure S4B).

Next, we investigated if the synergistic effect of ZFs or TALEs with CRISPR depended solely on the helicase/topoisomerase activity of dCas9 and/or the presence of targeted sgRNAs to recruit VP64 in the promoter region. We found that co-transfection of either CRISPR/dCas9 with no effector, or CRISPR/dCas9 VP64 without sgRNAs into HEK293T cells in combination with either ZFs (Supplementary Figure S4C) or TALEs (Supplementary Figure S4D) did not result in significant differences in mRNA expression compared to ZFs or TALEs transfected alone (individually or in combination, p>0.05). This indicated that the activation of a protein-based scaffold ATF was not improved by the helicase/topoisomerase activity of dCas9 in the absence of a transactivator. Moreover, the synergy between agents relied on sgRNAs to target multiple transactivators to the corresponding promoter.

Finally, we explored a combination of all three platforms (Figure 4C), transiently transfected into HEK293T cells in a ratio of 1:1:2 (ZFs/TALEs/CRISPR respectively). We found that co-transfecting all three platforms achieved the highest activation of *MASPIN* mRNA (1302.4-fold relative to control, p<0.0001) and protein re-expression. These results indicated that a combination of different protein-based molecular backbones enhanced the activation potential of CRISPR/dCas9, creating an opportunity for utilizing each platform with unique activator strategies.

**Figure 5:**
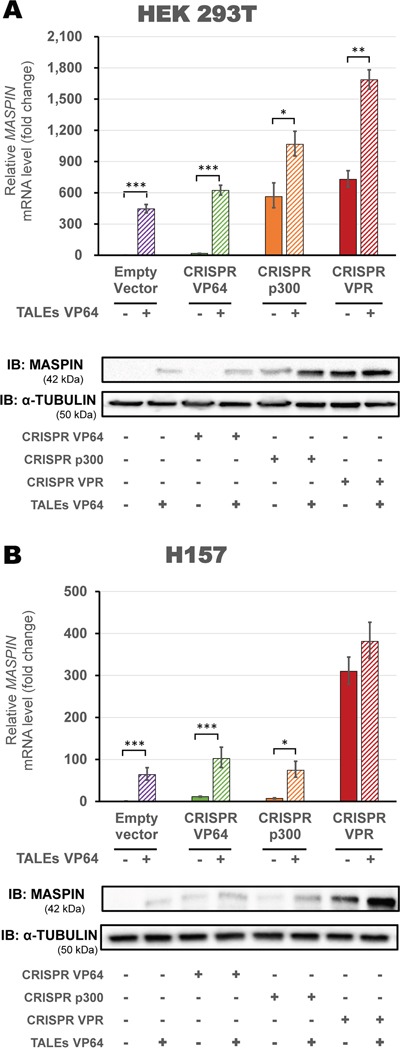
Up-regulation of MASPIN expression by combinations of different CRISPR/dCas9 constructs and protein-based DBD ATFs in highly methylated cell lines CRISPR/dCas9 systems using VP64 (green), p300 (orange) or VPR (red) and a mix of four gRNAs were transfected alone or in combination with both TALEs VP64 (**A** – HEK293T cells and **B** – H157 cells). qRT-PCR data indicate the fold change in *MASPIN* transcription levels relative to cells transfected with empty vector (pcDNA3.1 backbone), normalized against *GAPDH* expression (error bars represent ± SEM, n=3). Statistical significance: one-way ANOVA, corrected for multiple comparisons using the Tukey HSD test, p<0.05 (*), p<0.01 (**) and p<0.001 (***). Translation levels of MASPIN (42 kDa) and α-TUBULIN (50 kDa) as shown by a representative immunoblotting (n=2). The platforms delivered in each condition are indicated in the figure.

To maximize gene reactivation on highly methylated cell lines (HEK293T and H157), we transfected a panel of second-generation CRISPR/dCas9 activator domains in combination with TALEs. In HEK293T cells (Figure 5A), we found that activation was significantly enhanced when using CRISPR/dCas9 p300 or CRISPR/dCas9 VPR in combination with TALEs VP64 (1065.49-fold vs 563.36-fold relative to control, and 1685.21-fold vs 728.75-fold relative to control, when compared to CRISPR/dCas9 constructs alone, respectively, p<0.0001). Both combinations were significantly superior to the activation by TALEs alone (445.72-fold relative to control, p<0.0001). Protein levels showed a similar pattern of activation, with CRISPR/dCas9 VPR in combination with TALEs VP64 achieving the highest protein expression compared to all other combinations tested. In H157 cells (Figure 5B) we observed a significant increase in relative *MASPIN* mRNA levels in CRISPR/dCas9 VP64 or p300 cells co-transfected with TALEs (102.16-fold vs 11.41-fold relative to control, and 74.36-fold vs 7.28-fold relative to control, when compared to CRISPR/dCas9 constructs alone, respectively, p<0.0001). In contrast with VP64 or p300, co-transfection of CRISPR/dCas9 VPR with TALEs did not significantly increase the activation of *MASPIN* (381.28-fold vs 310.04-fold relative to control, when compared to CRISPR/dCas9 VPR alone, p>0.05).

**Figure 6:**
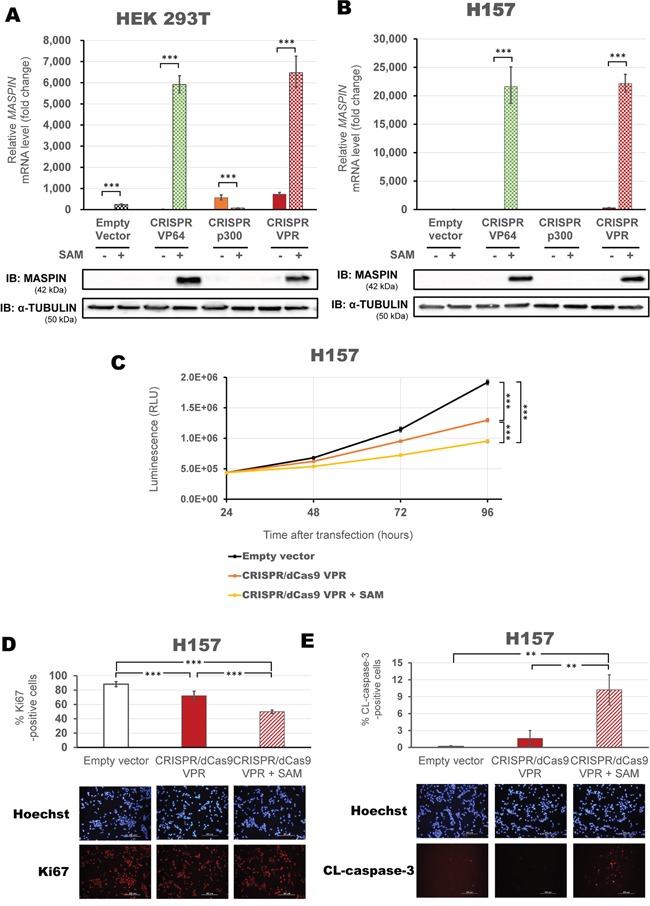
Maximizing activation of *MASPIN* expression using SAM complex correlates with phenotypic reprogramming CRISPR/dCas9 systems, linked to VP64 (green), p300 (orange) or VPR (red) were transfected using a mix of four modified gRNAs 2.0 in combination with the SAM complex MS2-p65-HSF1 helper proteins. HEK293T cells **A.** and H157 cells **B.** were transfected with or without the SAM complex for each effector domain fused to dCas9. qRT-PCR data indicate the fold change in *MASPIN* transcription levels relative to cells transfected with empty vector, normalized against *GAPDH* expression (error bars represent ± SEM, n=3). Statistical significance: one-way ANOVA, corrected for multiple comparisons using the Tukey HSD test, p<0.05 (*), p<0.01 (**) and p<0.0001 (***). Translation levels of MASPIN (42 kDa) and α-TUBULIN (50 kDa) as shown by a representative immunoblotting (n=2). **C.** Viability assay in H157 cells comparing three successful activation strategies using CRISPR/dCas9 VPR: alone (with a mix of four gRNAs, orange) or with the SAM complex (with a mix of four gRNAs 2.0, yellow), and compared to CRISPR/dCas9 No effector with non-specific gRNAs (empty vector, black). Data is shown in absolute luminescence (RLU) at 24, 48, 72 and 96 hours post transient transfection (error bars represent ± SEM, n=3), and comparison using t-student test with Welch's correction was performed to compare cell viability at 96 hours (***, p<0.0001). Immunofluorescence detection of Ki67-positive cells **D.** and cleaved caspase3 **E.** to assess proliferation and apoptosis in cells 96 hours post transfection with CRISPR/dCas9 VPR with or without SAM helper protein. Quantification is shown as percentage of corresponding positive cells and a representative image of Hoechst staining (blue) and either Ki67 or CL-caspase3 (red) are provided. Statistical significance: Student's t-test with Welch's correction, p<0.001 (**) and p<0.0001 (***).

To further explore combinatorial delivery of multiple effector functions, we next investigated the activation potential of the CRISPR/dCas9 and SAM complex [41]. When co-transfected into HEK293T cells (Figure 6A), the SAM helper protein enhanced *MASPIN* activation for both CRISPR/dCas9 VP64 and VPR constructs (5,916-fold and 6,477-fold relative to control, respectively, p<0.0001), but did not improve the effect of CRISPR/dCas9 p300 (p>0.05). Similarly, CRISPR/dCas9 VP64 and VPR activation was greatly enhanced in H157 cells by three orders of magnitude (21,640-fold and 22,145-fold relative to control, respectively, p<0.0001, Figure 6B), while CRISPR/dCas9 p300 activation was not enhanced by the addition of the SAM complex. MASPIN protein expression increased consistently with transcript upregulation in each cell line.

To correlate the extent of tumor suppression gene re-expression with cancer cell phenotypic reprogramming, we processed H157 cells transfected with CRISPR/dCas9 VPR alone or in association with SAM complex, and evaluated the impact on cell viability, proliferation and apoptosis. Consistently with the degree of MASPIN reactivation we found that CRISPR/dCas9 VPR with SAM was the most potent combination and significantly decreased cell viability relative to control (Figure 6C, p<0.0001), followed by CRISPR/dCas9 VPR alone, which in turn had a significantly lower cell survival rate than the control condition (p<0.0001). Consistently, we observed a decrease in expression of the proliferative marker Ki67 after 72 hours post transfection (50% vs 88% of Ki67-positive cells with CRISPR/dCas9 VPR + SAM compared to control, p<0.0001, Figure 6D). As expected from re-activation of MASPIN, an increase in cleaved caspase-3 staining by immunofluorescence validated induction of cell apoptosis associated with transfection of CRISPR/dCas9 VPR and SAM complex (10.22% vs 0.171% cleaved-caspase-3 positive cells compared to control, p<0.005, Figure 6E). In conclusion, these data suggested that a combinatorial approach to deliver multiple ATFs and effector domains maximized TSG re-expression, which correlated with functional tumor suppression and cancer cell phenotypic reprogramming.

### Validation of activation strategies on a novel class II TSG *REPRIMO*

**Figure 7:**
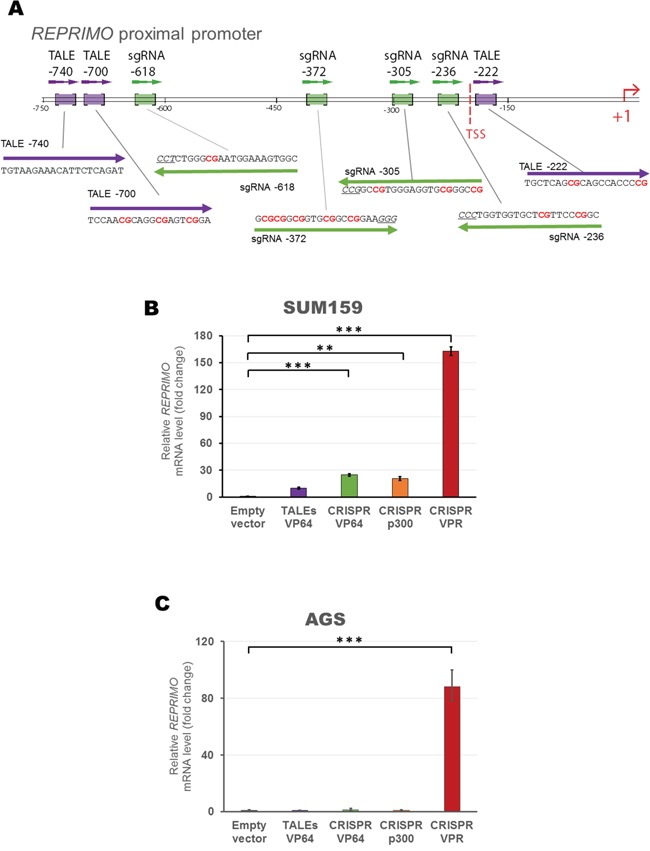
Design and construction of gRNAs and TALEs to activate *REPRIMO* mRNA expression in silenced cell lines **A.** Schematic representation of *REPRIMO* proximal promoter illustrating TALEs (purple) and gRNA (green) binding sites. TSS refers to transcription start site. qRT-PCR data showing *REPRIMO* expression after transient transfection of a combination of TALEs (purple) and CRISPR/dCas9 systems with a mix of four gRNAs (VP64 in green, p300 in orange and VPR in red) into SUM159 **B.** and AGS **C.** cells. Data indicate the fold change in *REPRIMO* transcription levels relative to cells transfected with empty vector, normalized against *GAPDH* expression (error bars represent ± SEM, n=3). Statistical significance: one-way ANOVA, corrected for multiple comparisons using the Tukey HSD test, p<0.01 (**) and p<0.0001 (***).

To extend and validate the previous findings in another gene context, we chose a gastric cancer class II TSG *REPRIMO*. We designed three TALEs and a set of four gRNAs targeted to the proximal promoter of *REPRIMO* (Figure 7A) and we first validated these constructs in a low-expressing *REPRIMO* cell line, the triple negative breast SUM159 line (Figure 7B). While CRISPR/dCas9 systems upregulated gene expression in varying degrees depending on the effector domains fused to dCas9 (24.7-, 20.3-, 162.6-fold relative to control using VP64, p300 or VPR, respectively, p<0.001), the mix of TALEs fused to VP64 did not upregulate significantly *REPRIMO* ‘s expression (9.7-fold relative to control, p>0.05). We next tested these constructs on a gastric cell line, AGS, which harbor a highly methylated *REPRIMO* promoter [55], and expressing very low basal levels of the endogenous gene. As it was observed in the case of *MASPIN* re-expression in H157 cells, AGS only showed a significant *REPRIMO* up-regulation with the CRISPR/dCas9 VPR construct (88.34-fold relative to control, p<0.0001, Figure 7C) whereas all the other individual ATFs tested failed to upregulate gene expression (p>0.05).

**Figure 8:**
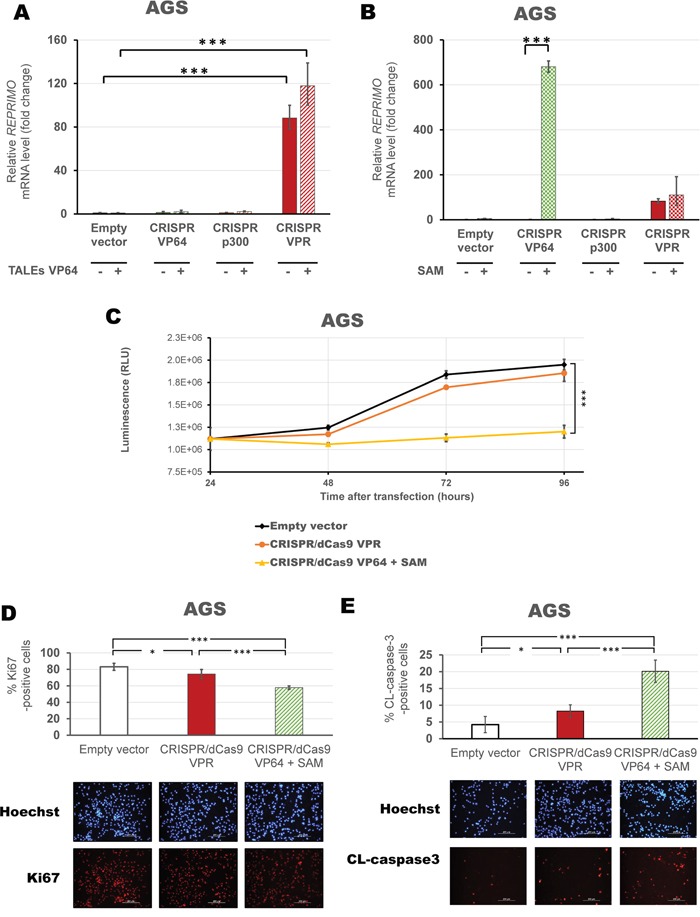
Activation of novel TSG, *REPRIMO*, using CRISPR/dCas9 + SAM complex induces changes in cell proliferation qRT-PCR data showing *REPRIMO* expression after transient transfection of CRISPR/dCas9 systems with a mix of four gRNAs (VP64 in green, p300 in orange and VPR in red) with or without a combination of TALEs **A.** or SAM complex (using a mix of four gRNAs 2.0) **B.** in AGS cells. Data indicate the fold change in *REPRIMO* transcription levels relative to cells transfected with empty vector (pcDNA3.1 backbone), normalized against *GAPDH* expression (error bars represent ± SEM, n=3). Statistical significance: one-way ANOVA, corrected for multiple comparisons using the Tukey HSD test, p<0.05 (*), p<0.01 (**) and p<0.0001 (***). **C.** Viability assay in AGS cells comparing three successful activation strategies using CRISPR/dCas9 VPR with four gRNAs (orange) or CRISPR/dCas9 VP64 with SAM complex and four gRNAs 2.0 (yellow), compared to CRISPR/dCas9 No effector with non-specific gRNAs (as empty vector in black). Data is shown in absolute luminescence (RLU) at 24, 48, 72 and 96 hours post transient transfection (error bars represent ± SEM, n=3), and comparison using t-student test with Welch's correction was performed to compare cell viability at 96 hours (***, p<0.0001). Immunofluorescence detection of Ki67-positive cells **D.** and cleaved caspase3 **E.** to assess proliferation and apoptosis in cells 96 hours post transfection with CRISPR/dCas9 VPR or CRISPR/dCas9 VP64 with SAM helper protein. Quantification is shown as percentage of corresponding positive cells and a representative image of Hoechst staining (blue) and either Ki67 or CL-caspase3 (red) are provided. Statistical significance: Student's t-test with Welch's correction, p<0.001 (**) and p<0.0001 (***).

Next, we evaluated the ability of the protein-based ATF, TALEs, in combination with CRISPR/dCas9 systems in AGS cells to improve reactivation of gene expression. CRISPR/dCas9 VPR further increased *REPRIMO* expression in combination with activating TALEs (117.94-fold relative to control, p<0.0001, Figure 8A), but was not significantly higher than CRISPR/dCas9 VPR alone (p>0.05). Finally, we co-transfected each individual CRISPR/dCas9 activating platforms (VP64/p300/VPR) with a set of gRNAs able to recruit the SAM complex (Figure 8B). We observed a significant enhancement of *REPRIMO* gene expression with both CRISPR/dCas9 VPR (110.44-fold relative to control, p<0.0001), and CRISPR/dCas9 VP64 (680.62-fold relative to control, p<0.0001) in association with the SAM helper protein. Accordingly, CRISPR/dCas9 VP64 with the SAM complex significantly inhibited gastric cancer cell viability as compared to empty vector and CRISPR/dCas9 VPR alone (p<0.0001, Figure 8C). A decrease in Ki67 positive cells (58% vs 83% Ki67-positive cells compared to control, p<0.0001, Figure 8D) and an increase in cleaved caspase3 positive cells (20.1% vs 4.2% cleaved-caspase-3 positive cells compared to control, p<0.0001, Figure 8E) demonstrated an inhibition of cell proliferation by induction of apoptosis associated with CRISPR/dCas9 VP64 with SAM.

In conclusion, the reactivation of two Class II TSGs suggested similar responses to epigenome editing platforms, which depended on the cell lines and DNA methylation states. CRISPR/dCas9 systems associated with the SAM complex consistently reactivated dormant TSGs in the cell lines with hypermethylated promoters. Further, the combination of activating TALEs and CRISPR/dCas9 platforms offers the possibility to incorporate mechanistically distinct epigenetic effector for the locus-specific reprogramming of the aberrant cancerous state.

## DISCUSSION

In this study, we have compared different epigenetic editing approaches involving combinations of CRISPR/dCas9 with protein-based ATFs (ZFs and TALEs). We used this combinatorial strategy to deliver state-of-the-art activator domains to effectively normalize TSG expression in cancerous cells. We have compared the potency of the three major ATF activator platforms across multiple cell lines with different epigenetic states and established robust synergisms between genome editing tools. We validated the use of combinatorial strategies using novel activator domains for powerful gene activation in a proof of concept TSG *MASPIN* and in the context of a novel TSG, *REPRIMO*, which are both silenced by DNA methylation in aggressive tumor cells. In both cases we found that the VPR domain and the SAM complex consistently enhanced upregulation with higher potency over all the other approaches investigated. Moreover, we reported for the first time a phenotypic reprogramming using transiently transfected CRISPR/dCas9 systems to re-activate TSG epigenetically silenced in cancer cells.

Although much work has been invested in the development of new platforms for gene activation, there is a scarcity of information comparing the efficacy of all platforms available. This is particularly important for epigenetically silenced TSGs, which require high levels of gene re-activation to elicit physiological and phenotypic responses. Here, we first compared ZFs, TALEs, and CRISPR/dCas9, equally engineered with the VP64 activator domain fused in the C-terminus of each protein. Despite the differences in size and number of plasmid components co-transfected in these experiments it was possible to evaluate the relative potency of single platforms and define synergistic combinations in reactivating gene expression. These synergistic interactions made possible the delivery of minimal amount of platforms in the combinations maximizing gene reactivation while decreasing potential toxicity.

Based on our group previous experience with ZFs activators successfully activating *MASPIN* [45, 49], we designed CRISPR and TALEs to bind in the same proximal promoter region that was validated as a hotspot for regulating its expression. We found that protein-based ATFs such as TALEs, were generally more potent as single agents than the CRISPR/dCas9 system in reactivating gene expression in the cell lines tested. Furthermore, we found that in cell lines having an epigenetically silenced gene, protein-based ATFs were highly synergistic with CRISPR/dCas9, while in cell lines having basal levels of gene expression CRISPR/dCas9 or TALEs as single agents were generally the most effective tool for gene reactivation. Wider tiling of the promoter with more gRNAs or protein DBDs is frequently recommended to enhance activation [61–63], but we also observed that by binding different scaffolds, even to the same locations, we significantly increased the effectiveness of gene upregulation. The enhancement of gene expression observed with ZFs and TALEs could be explained by remodeling of local chromatin configuration induced by binding of one individual platform, facilitating the binding of the second ATF. More work is required to determine the precise mechanism by which these ATFs could induce alterations in chromatin.

In an effort to enhance activation of gene expression of a target gene, novel effector domains have been developed and engineered in fusion with the CRISPR/dCas9 system. The classic activator domain VP64 recruits PC4, CBP/p300 and the SWI/SNF complex, while the alternative activator p65, a trans-activating subunit of NF-κB, present in VPR and SAM complex, recruits a different set of cofactors: AP-1, ATF/CREB and SP1. HSF1 is a novel activator from the human Heat-Shock Factor 1 (HSF1), and Rta (Replication and Transcription Activator) is also a potent activator domain, derived from the γ-herpesvirus family, that recruits cofactors involved in the immediate early transcription. On the other hand, p300 enhance activation indirectly by epigenetic reprogramming of histone marks [64], increasing H3K27 acetylation, an epigenetic mark associated to higher transcription activity, which could explain lower activation of gene expression in relevant cancer cell lines that likely lack endogenous drivers of TSGs, in contrast with a normal-like cell such as HEK293T. In the case of unmethylated promoters, the use of direct activators such as CRISPR/dCas9 VPR yielded the highest levels of mRNA and protein over all the other combinations tested.

We and others have demonstrated that combining these molecular tools can increase activation of gene expression [61, 62]. In order to overcome several epigenetics mechanisms silencing a TSG in a cancer cell, we used a combinatorial approach of CRISPR/dCas9 systems in addition to protein-based ATFs, in particular the use of TALEs was a strategy of choice based on their high specificity for target sequences. In addition, we exploited the SAM complex, which binds p65 and HSF1 to modified gRNAs that incorporate MS2 RNA aptamers into the stemloop and second loop which remain exposed in the ternary dCas9 complex [41]. Importantly, the SAM complex enhanced gene re-activation several orders of magnitude over other strategies tested. Interestingly, dCas9 VPR failed to improve activation over dCas9 VP64 when used together with the SAM complex, which could be explained by a possible conformational interference between large functional domains (VPR and SAM, VP64 being smaller in size) or by the fact that VP64 and SAM could recruit a set of non-overlapping and more effective co-activators present in the cells. Interestingly, the SAM complex failed to enhance or even inhibit p300 activation in all cell lines analyzed. Taken together, these data emphasized the importance of using the appropriate gene editing toolbox that is tailored to the cell line and epigenetic state of the cell, and confirmed the benefits of using multiple effectors to maximize gene expression for strongly silenced TSGs. More importantly, a phenotypic reprogramming was associated with higher levels of activation. Notably by using the SAM complex we observed a significant reduction in proliferation, diminished cell viability and increased apoptosis. Thus, we demonstrated a correlation between potent reactivation of TSG re-expression using transiently transfected ATFs and cell viability, proliferation and apoptosis.

Finally, the strategy validated in *MASPIN* as proof of concept was later extended in a novel TSG, *REPRIMO*. This gene is relevant in gastric cancer and the methylation status of its promoter has been proposed as an effective biomarker [57, 58, 65]. As a p53 dependent G2 arrest mediator, *REPRIMO*, has been demonstrated to have tumor suppressive functions when overexpressed [55, 59], however, regain of function using ATFs has not been previously achieved. We observed a marked reduction in proliferation when re-activating gene expression using CRISPR/dCas9 VP64 with SAM complex in a hyper methylated gastric cell line, AGS [55]. Once again, the combinatorial approach presented has proven consistently useful to induce phenotypic changes in cancer cells targeting otherwise silenced TSGs.

In summary, we have shown unprecedented reactivation of highly methylated and silenced TSGs using CRISPR/dCas9 systems. By exploiting combinatorial strategies of platforms and effector domains, we upregulated endogenous expression of relevant genes to control proliferation and apoptosis in cancer, thus showing a phenotypic reprogramming *via* non-viral delivery of novel molecular tools for epigenome editing. Epigenetic modifying agents, such as DNA demethylating molecules [66] (e.g., the drug Decitabine [67], a 5-aza-2′-deoxycytidine) or Histone deacetyltransferase's inhibitors [68] (e.g., the potent inhibitor Suberoylanilide hydroxamic acid, SAHA), have shown positive clinical results on hematological cancer, as well as some solid tumors [5, 69]. However, the mechanisms are not fully understood and risks of adverse and unpredictable side effects are a concern due to the unspecific nature of these drugs [46, 70]. Here we proposed a more targeted strategy to modify the epigenetic and gene expression patterns of cancer cells to reprogram their naïve diseased phenotype. If successful, this strategy could overcome the toxicity associated with current drugs and collateral side effects due to genome-wide modifications, although efficient and non-toxic delivery methods still remain a challenge. In addition to testing novel alternatives to deliver CRISPR systems to cancer cells, the next step is to perform *in vivo* studies to determine the impact of waking up dormant TSGs in a growing tumor in animal models.

In conclusion, we have systematically compared some of the available ATF platforms for epigenome editing (ZFs, TALEs and CRISPR/dCas9) of two silenced tumor suppressor genes (*MASPIN* and *REPRIMO*) to induce potent reactivation of endogenous gene expression. Moreover, we observed that each cell line responded differently to a particular effector domain or combination of ATFs and that therefore the repertoire of platforms and effectors must be carefully optimized for each gene and epigenetic state of the cell line tested. In addition, we compared more recently characterized state-of-the-art activator domains, including p300, VPR and the SAM complex, investigating the potential to reactivate TSG expression as single platforms and in combinations with protein-based ATFs. More importantly, we showed that non-viral delivery of CRISPR/dCas9 systems, especially in combination with the SAM complex, produced a robust activation of target dormant TSGs and effectively awaken them, thus regaining their suppressive functions, as seen by a decrease in proliferation rates and increase in apoptosis. These findings provide proof of concept to exploit these technologies, CRISPR/dCas9 systems in particular, on other dormant TSGs, restoring their functions to reprogram a cancerous phenotype. Non-viral delivery of ATFs to modulate gene expression remains a challenge for an effective therapy in cancer patients, however, our study provides evidence for future applications of the CRISPR/dCas9 technology as a transient, targeted and efficient tool to restore tumor suppressive functions of dormant genes in cancer cells.

## MATERIALS AND METHODS

### Artificial transcription factor constructs

ZF fused with effector domain VP64. ZF-97 and ZF-126 with VP64 have been described previously [45]. TALE fused with effector domain VP64. TALE-99 and TALE-128 with VP64 were designed to target the same sites as ZF-97 and ZF-126, respectively. Each TALE targets two more nucleotides than its corresponding ZF (a total of 20 nt) in order to start in the nearest thiamine nucleotide. The custom-designed sequence was engineered by Genecopoeia TALE-TF service.

CRISPR/dCas9 fused with effector domains VP64/p300/VPR. All dCas9 constructs used an inactivated form of *S. pyogenes* Cas9 protein with two mutations in D10A and H840A. pcDNA3.1-dCas9-VP64 and pcDNA3.1-dCas9-p300 (Addgene plasmid # 47107 and 61357, respectively) had a C-terminal fusion of VP64 domain or human p300 HAT core (aa 1048-1664) domain [39], respectively, driven by a CMV promoter, a gift from Charles Gersbach. The pcDNA-dCas9-No Effector is HA tagged at the C-terminus (Addgene plasmid # 47106). pcDNA-dCas9-VPR [40] with VP64, p65 and Rta fused to its C-terminus (Addgene plasmid 63798) was a gift from George Church. All dCas9 vectors used the same set of four sgRNAs based on the pSP-gRNA backbone vector (Addgene plasmid # 47108). Individual guides were designed using the crispr.mit.edu website tool to find target sequences in *MASPIN* and *REPRIMO* proximal promoters (Supplementary Figure S1) and cloned into BbsI sites in the sgRNA expression vector, pSP-gRNA backbone, following Gersbach's protocol [24].

### Cell lines, cell culture and transfection

The HEK293T human embryonic kidney cell line was obtained from the American Type Culture Collection (ATCC, Manassas). The H157 human lung cancer cell line was kindly provided by Dr. Robert Winn (University of Colorado Health Science Center). Human breast cancer cell lines MCF7 and SUM159 were obtained from the Tissue Culture Facility of the UNC Lineberger Comprehensive Cancer Center (University of North Carolina, Chapel Hill). SUM159 cells were cultured in F12 media supplemented with 5% FBS, 5 μg/mL insulin, 1 μg/mL hydrocortisone. MCF7 cells were cultured in MEM alpha media supplemented with 10% FBS and 1% each of sodium pyruvate, sodium bicarbonate and non-essential amino acids. HEK293T cells were cultured in DMEM media supplemented with 10% FBS. H157 cells were cultured in RPMI 1640 supplemented with 5% FBS. All cells were cultured with 1% penicillin/streptomycin unless stated otherwise.

Transfections were performed in 6-well plates using Lipofectamine 2000 reagent (Invitrogen) according to the manufacturer's protocol. 5 x10^5^ cells were seeded 24 hours prior to transfection in their corresponding media, washed with PBS and cultured in Opti-MEM (750 μL). Each well was transfected using 4.8 μl of Lipofectamine 2000 in 250 μL of Opti-MEM with 2.4 μg of total plasmid DNA, according to the manufacturer's protocol. All transfections had the same total amount of plasmid DNA and pcDNA3.1 empty vector was added to complete the total amount of 2.4 μg per well. Genomic DNA, total RNA and protein were collected 48 hours post-transfection. The transfection efficiency of these combinations in H157 cells was between 40-50%, as assessed by flow cytometry of a control EGFP plasmid.

### Methylation assay

Genomic DNA was obtained using QIAamp DNA Mini and Blood Mini Kit (Qiagen), as per manufacturer's instructions, in wild type cells. All methylation assays were done by pyrosequencing designed using the algorithms built into the PyroMark Assay Design Software (Version 2.0.1, Qiagen). *MASPIN* primers sequences used are as follows: forward PCR primer, TGGATAAGTTGTTAAGAGGTTTGAGTAG, reverse PCR primer, 5′Biotin-ACTACCCCACCTTACTTACCTA, and sequencing primers, S1-GTTTGAGTAGGAGAGGA and S2-GTGTTGTTTAGGTGAGTTA. DNA samples were converted using the Epitect Bisulphite Conversion Kit (Qiagen). 500 ng of genomic DNA were converted overnight in 140 μL total volume using the standard protocol from the kit.

All PCR amplifications were performed with the PyroMark PCR Kit (Qiagen), as per manufacturer's instructions. The PCR product was bound to Streptavidin Sepharose High Performance beads (GE Healthcare Life Sciences), the beads containing the immobilized PCR product were denatured and washed using proprietary solutions (Qiagen) on the Pyrosequencing Vacuum Prep Tool (Qiagen) to isolate a single stranded template. The beads were then transferred to an optically clear, 24 well sequencing plate in 0.3 μM of pyrosequencing primer. Annealing to the single-stranded template was done by heating the plate to 80°C followed by cooling to room temperature. Pyrosequencing was performed on a PyroMark 24 Pyrosequencing System (Qiagen) as per the manufacturer's instructions. Data was analyzed on the PyroMark Q24 software to give the %methylation values for each CpG site in the sample.

### RNA extraction, cDNA conversion and relative qPCR

RNA was extracted from transfected cells using QIAzol Lysis Reagent (QIAGEN) according to manufacturer's instructions, and 2 μg of purified total RNA was used as the template for cDNA synthesis using the High Capacity cDNA Reverse Transcription Kit (Applied Biosystems). Relative quantification of transcript expression (*MASPIN*, *REPRIMO* and *GAPDH*) was obtained by quantitative real-time PCR (qRT-PCR) using commercially available fluorescent TaqMan probes (Applied Biosystems) in the ViiA 7 Real-Time PCR machine (Applied Biosystems), and analyzed using QuantStudio Real Time PCR Software (v1.1, Applied Biosystems). Data were analyzed according to MIQE guidelines [71] and results are expressed as fold change compared to empty vector transfected cells after normalization against *GAPDH* mRNA levels.

### Western blotting

Cell lysates were collected using Cell Lysis Buffer 1x (Cell Signaling) supplemented with 1 mM PMSF and sonicated for 5 seconds at 10 mA. From each sample, 65 μg of protein was resolved by 10% SDS/PAGE (BioRad) and subsequently transferred to a PVDF membrane (BioRad). Immunoreactivity was determined with mouse anti-MASPIN (monoclonal, 1: 1,000 dilution, Pharmingen) or with mouse anti-alpha-TUBULIN (monoclonal, 1: 5,000 dilution, clone B512, Sigma-Aldritch) primary antibodies and goat anti-MOUSE-HRP secondary antibody (1:10,000 dilution, GE Technology). Visualization was performed by enhanced chemiluminescence, Novex ECL Chemiluminescent Substrate Reagent Kit (Invitrogen) with the ChemiDoc MP system (BioRad). All membrane pictures were digitally obtained in the ChemiDoc MP system and processed using ImageLab Software (v5.2, BioRad) and ImageJ.

### Cell viability

Cell viability for H157 and AGS cells was assessed using CellTiter-Glo 2.0 Assay (Promega, NSW, Australia), according to the manufacturer's protocol, and luminescence was measured using the EnVision 2102 Multilabel Reader (PerkinElmer; Waltham, MA, USA). In brief, cells were seeded on a 6-well plate and transfected 24 hours later using Lipofectamine 2000 (Invitrogen). After a 24 hours' incubation, cells were collected using trypsin-EDTA 0.25% and seeded in 96-well plate wells (4 replicates plus one background control). Measurements were made at 24, 48, 72 and 96 hours' intervals post transfection. Data (three biological replicates) were normalized to the average luminescence at 24 hours (first data point) and presented as luminescence RLU.

### Immunofluorescence assay

Apoptosis and proliferation were assessed by immunofluorescence visualization of cleaved caspase-3 (CL-caspase3) and Ki-67-positive cells, respectively, as previously described [72]. H157 and AGS cells were transfected in 6-well plates with different combinations of plasmids using Lipofectamine 2000 (Invitrogen) and collected 24 hours afterwards to be distributed in Lab-Tek Chamber slides system, 2×10^5^ cells per chamber. After 72 hours of transfection, cells were fixed with 4% para formaldehyde for 20 minutes, washed with PBS, blocked with blocking solution (5% fetal bovine serum, 0.3% Triton X-100 in PBS) for 1 hour, incubated with anti-CL caspase3 primary antibody (1:400 dilution; Cell Signaling Technology, QLD, Australia) or anti-Ki67 (1:400 dilution; Cell Signaling Technology, QLD, Australia) in blocking solution overnight and further incubated with an anti-rabbit or anti-mouse (for CL caspase3 or Ki67, respectively) secondary antibody Alexa Fluor 594-conjugated antibody (1:1000 dilution; Cell Signaling Technology, QLD, Australia) and Hoechst 33258 nucleic staining (1:10000 dilution). The percentage of positive cells was determined by counting red fluorescent cells versus total cells using a fluorescent microscope (Olympus IX71).

### Statistical analysis

Statistical analyses were performed with GraphPad Prism v6 (GraphPad Software Inc.) and Office Excel 365 (Microsoft). Statistical significance was determined using an unpaired one-way ANOVA with the Tukey HSD post hoc test correcting for multiple comparisons for qRT-PCR data, or unpaired two sample Student's *t*-tests (two-sided) for functional assays (viability, proliferation or apoptosis). For all tests, differences were considered significant at p<0.05 (*), p<0.001 (**) and p<0.0001 (***).

## SUPPLEMENTARY FIGURES AND TABLE



## References

[1] Portela A, Esteller M (2010). Epigenetic modifications and human disease. Nat Biotechnol.

[2] Kazanets A, Shorstova T, Hilmi K, Marques M, Witcher M (2016). Epigenetic silencing of tumor suppressor genes: Paradigms, puzzles and potential. Biochim. Biophys. Acta - Rev. Cancer.

[3] Yen C-Y, Huang H-W, Shu C-W, Hou M-F, Yuan S-SF, Wang H-R, Chang Y-T, Farooqi AA, Tang J-Y, Chang H-W (2016). DNA methylation, histone acetylation and methylation of epigenetic modifications as a therapeutic approach for cancers. Cancer Lett.

[4] Kelly TK, De Carvalho DD, Jones PA (2010). Epigenetic modifications as therapeutic targets. Nat Biotechnol.

[5] Mei Q, Chen M, Lu X, Li X, Duan F, Wang M, Luo G, Han W (2015). An open-label, single-arm, phase I/II study of lower-dose decitabine based therapy in patients with advanced hepatocellular carcinoma. Oncotarget.

[6] Dawson M a, Kouzarides T (2012). Cancer epigenetics: From mechanism to therapy. Cell.

[7] Falahi F, Sgro A, Blancafort P (2015). Epigenome Engineering in Cancer: Fairytale or a Realistic Path to the Clinic?. Front. Oncol.

[8] Ansari AZ, Mapp AK (2002). Modular design of artificial transcription factors. Curr. Opin. Chem. Biol.

[9] Van Tol N, Van Der Zaal BJ (2014). Review: Artificial transcription factor-mediated regulation of gene expression. Plant Sci.

[10] Beerli RR, Barbas CF (2002). Engineering polydactyl zinc-finger transcription factors. Nat Biotechnol.

[11] Maeder ML, Linder SJ, Reyon D, Angstman JF, Fu Y, Sander JD, Joung JK (2013). Robust, synergistic regulation of human gene expression using TALE activators. Nat Methods.

[12] La Russa MF, Qi LS (2015). The New State of the Art: Cas9 for Gene Activation and Repression. Mol Cell Biol.

[13] Emerson RO, Thomas JH (2009). Adaptive evolution in zinc finger transcription factors. PLoS Genet.

[14] Kang JS, Kim JS (2000). Zinc finger proteins as designer transcription factors. J. Biol. Chem.

[15] Laity JH, Lee BM, Wright PE (2001). Zinc finger proteins: New insights into structural and functional diversity. Curr. Opin. Struct. Biol.

[16] Grimmer MR, Stolzenburg S, Ford E, Lister R, Blancafort P, Farnham PJ (2014). Analysis of an artificial zinc finger epigenetic modulator: widespread binding but limited regulation. Nucleic Acids Res.

[17] Huisman C, van der Wijst MG, Falahi F, Overkamp J, Karsten G, Terpstra MM, Kok K, van der Zee AG, Schuuring E, Wisman GBA, Rots MG (2015). Prolonged re-expression of the hypermethylated gene EPB41L3 using artificial transcription factors and epigenetic drugs. Epigenetics.

[18] Crocker J, Stern DL (2013). TALE-mediated modulation of transcriptional enhancers *in vivo*. Nat. Methods.

[19] Gao X, Yang J, Tsang JCH, Ooi J, Wu D, Liu P (2013). Reprogramming to pluripotency using designer TALE transcription factors targeting enhancers. Stem Cell Reports.

[20] Hockemeyer D, Wang H, Kiani S, Lai CS, Gao Q, Cassady JP, Cost GJ, Zhang L, Santiago Y, Miller JC, Zeitler B, Cherone JM, Meng X (2011). Genetic engineering of human pluripotent cells using TALE nucleases. Nat. Biotechnol.

[21] Clark KJ, Voytas DF, Ekker SC (2011). A TALE of Two Nucleases: Gene Targeting for the Masses?. Zebrafish.

[22] Morbitzer R, Römer P, Boch J, Lahaye T (2010). Regulation of selected genome loci using de novo-engineered transcription activator-like effector (TALE)-type transcription factors. Proc. Natl. Acad. Sci. U. S. A.

[23] Zhang F, Cong L, Lodato S, Kosuri S, Church GM, Arlotta P (2011). Efficient construction of sequence-specific TAL effectors for modulating mammalian transcription. Nat. Biotechnol.

[24] Kabadi AM, Gersbach C a (2014). Engineering synthetic TALE and CRISPR/Cas9 transcription factors for regulating gene expression. Methods Elsevier Inc.

[25] Deng D, Yan C, Wu J, Pan X, Yan N (2014). Revisiting the TALE repeat. Protein Cell.

[26] Deng D, Yan C, Pan X, Mahfouz M, Wang J, Zhu J-K, Shi Y, Yan N (2012). Structural basis for sequence-specific recognition of DNA by TAL effectors. Science.

[27] Boch J, Scholze H, Schornack S, Landgraf A, Hahn S, Kay S, Lahaye T, Nickstadt A, Bonas U (2009). Breaking the code of DNA binding specificity of TAL-type III effectors. Science.

[28] Moscou MJ, Bogdanove AJ (2009). A simple cipher governs DNA recognition by TAL effectors. Science.

[29] Holkers M, Maggio I, Liu J, Janssen JM, Miselli F, Mussolino C, Recchia A, Cathomen T, Goncalves MAF V, Gonçalves M a F V (2013). Differential integrity of TALE nuclease genes following adenoviral and lentiviral vector gene transfer into human cells. Nucleic Acids Res.

[30] Sanjana NE, Cong L, Zhou Y, Cunniff MM, Feng G, Zhang F (2012). A transcription activator-like effector toolbox for genome engineering. Nat Protoc.

[31] Jinek M, Chylinski K, Fonfara I, Hauer M, Doudna JA, Charpentier E (2012). A Programmable Dual-RNA – Guided DNA Endonuclease in Adaptive Bacterial Immunity. Science (80-).

[32] Esvelt KM, Mali P, Braff JL, Moosburner M, Yaung SJ, Church GM (2013). Orthogonal Cas9 proteins for RNA-guided gene regulation and editing. Nat Methods.

[33] Doudna JA, Charpentier E (2014). The new frontier of genome engineering with CRISPR-Cas9. Science (80-).

[34] Sternberg SH, Redding S, Jinek M, Greene EC, Doudna JA (2014). DNA interrogation by the CRISPR RNA-guided endonuclease Cas9. Nature Nature Publishing Group.

[35] Qi LS, Larson MH, Gilbert L a, Doudna J a, Weissman JS, Arkin AP, Lim W a (2013). Repurposing CRISPR as an RNA-guided platform for sequence-specific control of gene expression. Cell Elsevier.

[36] Bikard D, Jiang W, Samai P, Hochschild A, Zhang F, Marraffini LA (2013). Programmable repression and activation of bacterial gene expression using an engineered CRISPR-Cas system. Nucleic Acids Res.

[37] Perez-Pinera P, Kocak DD, Vockley CM, Adler AF, Kabadi AM, Polstein LR, Thakore PI, Glass KA, Ousterout DG, Leong KW, Guilak F, Crawford GE, Reddy TE (2013). RNA-guided gene activation by CRISPR-Cas9–based transcription factors. Nat. Methods.

[38] Farzadfard F, Perli SD, Lu TK (2013). Tunable and Multifunctional Eukaryotic Transcription Factors Based on CRISPR/Cas. ACS Synth. Biol.

[39] Hilton IB, D'Ippolito AM, Vockley CM, Thakore PI, Crawford GE, Reddy TE, Gersbach CA (2015). Epigenome editing by a CRISPR-Cas9-based acetyltransferase activates genes from promoters and enhancers. Nat. Biotechnol.

[40] Chavez A, Scheiman J, Vora S, Pruitt BW, Tuttle M, P R Iyer E, Lin S, Kiani S, Guzman CD, Wiegand DJ, Ter-Ovanesyan D, Braff JL, Davidsohn N (2015). Highly efficient Cas9-mediated transcriptional programming. Nat. Methods.

[41] Konermann S, Brigham MD, Trevino AE, Joung J, Abudayyeh OO, Barcena C, Hsu PD, Habib N, Gootenberg JS, Nishimasu H, Nureki O, Zhang F (2015). Genome-scale transcriptional activation by an engineered CRISPR-Cas9 complex. Nature.

[42] Berardi R, Morgese F, Onofri A, Mazzanti P, Pistelli M, Ballatore Z, Savini A, De Lisa M, Caramanti M, Rinaldi S, Pagliaretta S, Santoni M, Pierantoni C (2013). Role of maspin in cancer. Clin. Transl. Med.

[43] Machowska M, Wachowicz K, Sopel M, Rzepecki R (2014). Nuclear location of tumor suppressor protein maspin inhibits proliferation of breast cancer cells without affecting proliferation of normal epithelial cells. BMC Cancer.

[44] Huisman C, van der Wijst MGP, Schokker M, Blancafort P, Terpstra MM, Kok K, van der Zee AGJ, Schuuring E, Wisman GBA, Rots MG (2015). Re-expression of selected epigenetically silenced candidate tumor suppressor genes in cervical cancer by TET2-directed demethylation. Mol. Ther.

[45] Beltran A, Parikh S, Liu Y, Cuevas BD, Johnson GL, Futscher BW, Blancafort P (2007). Re-activation of a dormant tumor suppressor gene maspin by designed transcription factors. Oncogene.

[46] Beltran AS, Sun X, Lizardi PM, Blancafort P (2008). Reprogramming epigenetic silencing: artificial transcription factors synergize with chromatin remodeling drugs to reactivate the tumor suppressor mammary serine protease inhibitor. Mol. Cancer Ther.

[47] Lara H, Wang Y, Beltran AS, Juárez-Moreno K, Yuan X, Kato S, Leisewitz A V, Cuello Fredes M, Licea AF, Connolly DC, Huang L, Blancafort P (2012). Targeting serous epithelial ovarian cancer with designer zinc finger transcription factors. J. Biol. Chem.

[48] Beltran AS, Russo A, Lara H, Fan C, Lizardi PM, Blancafort P (2011). Suppression of breast tumor growth and metastasis by an engineered transcription factor. PLoS One.

[49] Beltran AS, Blancafort P (2011). Reactivation of MASPIN in non-small cell lung carcinoma (NSCLC) cells by artificial transcription factors (ATFs). Epigenetics.

[50] Rivenbark AG, Stolzenburg S, Beltran AS, Yuan X, Rots MG, Strahl BD, Blancafort P (2012). Epigenetic reprogramming of cancer cells via targeted DNA methylation. Epigenetics.

[51] Kwon Y-J, Lee SJ, Koh JS, Kim SH, Lee HW, Kang MC, Bae JB, Kim Y-J, Park JH (2012). Genome-wide analysis of DNA methylation and the gene expression change in lung cancer. J. Thorac. Oncol. International Association for the Study of Lung Cancer.

[52] Wang X, Wang Y, Li SL, Dong B, Zheng QF, Yan S, Ma YY, Zhang JZ, Fang J, Wu N, Wu HJ, Yang Y (2014). Decreased maspin combined with elevated vascular endothelial growth factor C is associated with poor prognosis in non-small cell lung cancer. Thorac. Cancer.

[53] Lonardo F, Li X, Kaplun A, Soubani A, Sethi S, Gadgeel S, Sheng S (2010). The natural tumor suppressor protein maspin and potential application in non small cell lung cancer. Curr. Pharm. Des.

[54] Ohki R, Nemoto J, Murasawa H, Oda E, Inazawa J, Tanaka N, Taniguchi T (2000). Reprimo, a new candidate mediator of the p53-mediated cell cycle arrest at the G2 phase. J. Biol. Chem.

[55] Saavedra K, Valbuena J, Olivares W, Marchant MJ, Rodríguez A, Torres-Estay V, Carrasco-Avino G, Guzmán L, Aguayo F, Roa JC, Corvalán AH (2015). Loss of Expression of Reprimo, a p53-induced Cell Cycle Arrest Gene, Correlates with Invasive Stage of Tumor Progression and p73 Expression in Gastric Cancer. PLoS One.

[56] Ooki A, Yamashita K, Yamaguchi K, Mondal A, Nishimiya H, Watanabe M (2013). DNA damage-inducible gene, reprimo functions as a tumor suppressor and is suppressed by promoter methylation in gastric cancer. Mol. Cancer Res.

[57] Sandoval-Bórquez A, Saavedra K, Carrasco-Avino G, Garcia-Bloj B, Fry J, Wichmann I, Corvalán AH (2015). Noncoding Genomics in Gastric Cancer and the Gastric Precancerous Cascade: Pathogenesis and Biomarkers. Dis. Markers.

[58] Bernal C, Aguayo F, Villarroel C, Vargas M, Díaz I, Ossandon FJ, Santibáñez E, Palma M, Aravena E, Barrientos C, Corvalan AH (2008). Reprimo as a potential biomarker for early detection in gastric cancer. Clin. Cancer Res.

[59] Xu M, Knox AJ, Michaelis KA, Kiseljak-Vassiliades K, Kleinschmidt-DeMasters BK, Lillehei KO, Wierman ME (2012). Reprimo (RPRM) is a novel tumor suppressor in pituitary tumors and regulates survival, proliferation, and tumorigenicity. Endocrinology.

[60] Chou TC (2010). Drug combination studies and their synergy quantification using the chou-talalay method. Cancer Res.

[61] Gao X, Tsang JCH, Gaba F, Wu D, Lu L, Liu P (2014). Comparison of TALE designer transcription factors and the CRISPR/dCas9 in regulation of gene expression by targeting enhancers. Nucleic Acids Res.

[62] Hu J, Lei Y, Wong W-KW-K, Liu S, Lee K-CK-C, He X, You W, Zhou R, Guo J-TJ-T, Chen X, Peng X, Sun H, Huang HH (2014). Direct activation of human and mouse Oct4 genes using engineered TALE and Cas9 transcription factors. Nucleic Acids Res.

[63] Perez-Pinera P, Ousterout DG, Brunger JM, Farin AM, Glass KA, Guilak F, Crawford GE, Hartemink AJ, Gersbach CA (2013). Synergistic and tunable human gene activation by combinations of synthetic transcription factors. Nat. Methods.

[64] Delvecchio M, Gaucher J, Aguilar-Gurrieri C, Ortega E, Panne D (2013). Structure of the p300 catalytic core and implications for chromatin targeting and HAT regulation. Nat. Struct. Mol. Biol.

[65] Riquelme I, Saavedra K, Espinoza JA, Weber H, Garcia P, Nervi B, Garrido M, Corvalan AH, Roa JC, Bizama C (2015). Molecular classification of gastric cancer: Towards a pathway-driven targeted therapy. Oncotarget.

[66] Nie J, Zhang Y, Li X, Chen M, Liu C, Han W (2016). DNA demethylating agent decitabine broadens the peripheral T cell receptor repertoire. Oncotarget.

[67] Nie J, Liu L, Li X, Han W (2014). Decitabine, a new star in epigenetic therapy: the clinical application and biological mechanism in solid tumors. Cancer Lett.

[68] Hegde M, Mantelingu K, Pandey M, Pavankumar CS, Rangappa KS, Raghavan SC (2016). Combinatorial Study of a Novel Poly (ADP-ribose) Polymerase Inhibitor and an HDAC Inhibitor, SAHA, in Leukemic Cell Lines. Target. Oncol.

[69] Fan H, Lu X, Wang X, Liu Y, Guo B, Zhang Y, Zhang W, Nie J, Feng K, Chen M, Zhang Y, Wang Y, Shi F (2014). Low-dose decitabine-based chemoimmunotherapy for patients with refractory advanced solid tumors: a phase I/II report. J. Immunol. Res.

[70] Cowan LA, Talwar S, Yang AS (2010). Will DNA methylation inhibitors work in solid tumors? A review of the clinical experience with azacitidine and decitabine in solid tumors. Epigenomics.

[71] Huggett JF, Foy CA, Benes V, Emslie K, Garson JA, Haynes R, Hellemans J, Kubista M, Mueller RD, Nolan T, Pfaffl MW, Shipley GL, Vandesompele J (2013). The digital MIQE guidelines: Minimum Information for Publication of Quantitative Digital PCR Experiments. Clin. Chem.

[72] Sorolla A, Ho D, Wang E, Evans CW, Ormonde CFG, Rashwan R, Singh R, Iyer KS, Blancafort P (2016). Sensitizing basal-like breast cancer to chemotherapy using nanoparticles conjugated with interference peptide. Nanoscale Royal Society of Chemistry.

