# Supplementary material for: Waking up dormant tumor suppressor genes with zinc fingers, TALEs and the CRISPR/dCas9 system
Source: Oncotarget. 2016 Aug 9;7(37):60535–54. doi: 10.18632/oncotarget.11142 (PMC5312401; doi:10.18632/oncotarget.11142)
Supplement: Supplementary file 1 [file oncotarget-07-60535-s001.pdf]

Waking up dormant tumor suppressor genes with zinc fingers, TALEs and the CRISPR/dCas9 system

SUPPLEMENTARY FIGURES AND TABLE

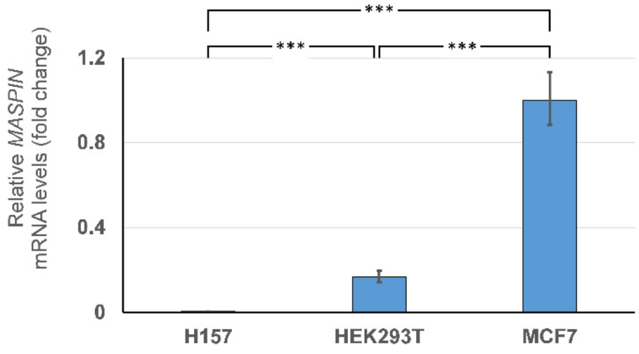

Supplementary Figure S1: Basal expression of MASPIN in three different cell lines. qRT-PCR showing MASPIN mRNA expression levels in MCF7, HEK293T and H157 cells indicated as fold change normalized to GAPDH mRNA expression levels and relative to MCF7 (error bars represent ± SEM, n=3). Statistical significance: one-way ANOVA, corrected for multiple comparisons using the Tukey HSD test, $p<0.0001$ (***).

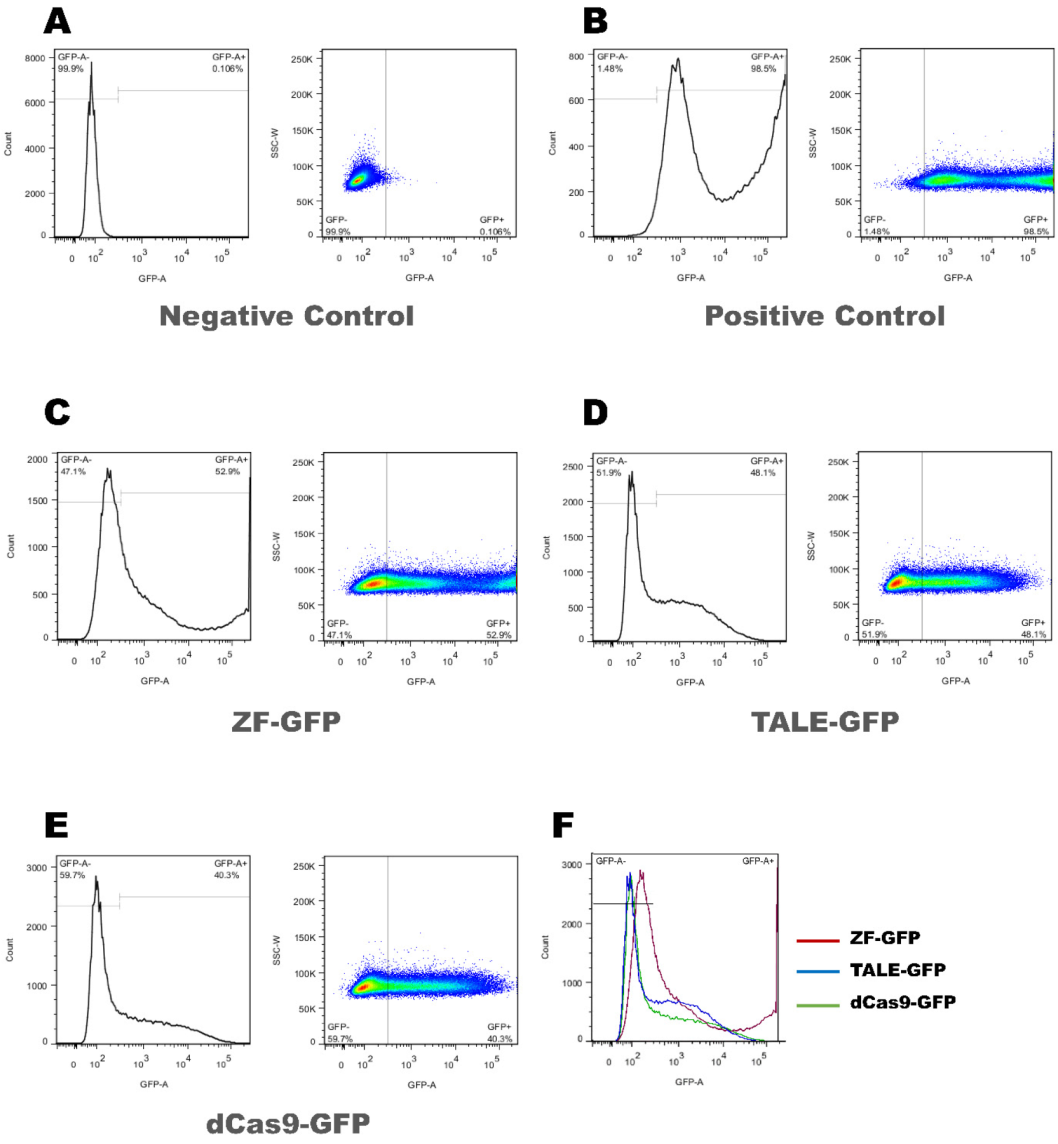

G

Construct	Percentage of GFP-positive cells
ZF-GFP	52.7 ± 0.4 %
TALE-GFP	47.0 ± 1.6 %
dCas9-GFP	40.3 ± 3.3 %

Supplementary Figure S2: Protein-based ATFs and CRISPR/dCas9 systems have similar transfection efficiency. Each construct, C-terminally fused to GFP was transfected into HEK293T cells and transfection efficiency was measured by flow cytometry. Positive cells were determined using a negative control (empty vector) **A.** and a positive control (eGFP N1) **B.** Percentage of positive cells is shown for ZF-GFP **C.** TALE-GFP **D.** and dCas9-GFP **E.** and **F.** An overlay of ZF (in red), TALE (in blue) and dCas9 (green) intensity is shown for comparison. **G.** Table showing average percentage of positive cells ± SD for each construct.

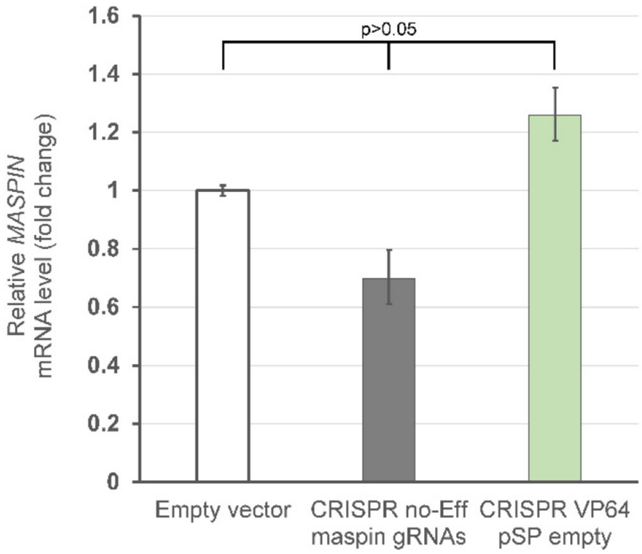

Supplementary Figure S3: CRISPR/dCas9 requires sgRNAs and an effector domain to activate MASPIN expression. qRT-PCR showing MASPIN expression in HEK293T cells after transfection with empty vector, dCas9 No Effector (CRISPR no-Eff) with MASPIN gRNAs (maspin gRNAs), or dCas9 VP64 (CRISPR VP64) without MASPIN gRNAs (pSP empty). Data indicate the fold change in MASPIN transcription levels relative to cells transfected with empty vector, normalized against GAPDH expression (error bars represent ± SEM, n=3). Statistical significance: one-way ANOVA, corrected for multiple comparisons using the Tukey HSD test, $p>0.05$ means differences were non-significant.

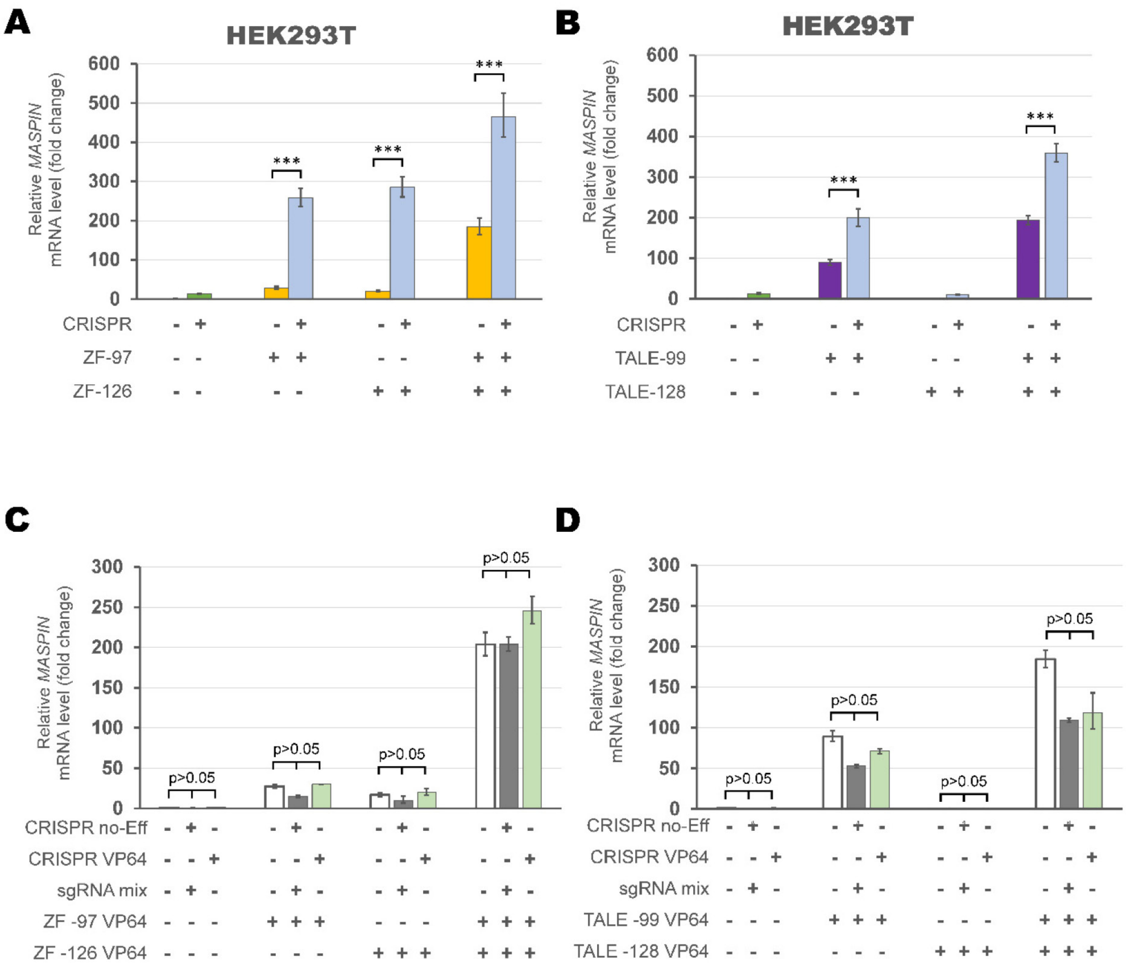

Supplementary Figure S4: Individual protein-based ATFs enhances CRISPR/dCas9 activation of MASPIN expression. *MASPIN* mRNA expression levels after transient transfection of CRISPR/dCas9 VP64 with ZFs VP64 **A.** and TALE VP64 **B.** as individual agents. qRT-PCR showing MASPIN expression after transfection with CRISPR/dCas9 VP64 alone (in green), ZFs (in yellow, A) or TALEs (in purple, B) and combinations of CRISPR/dCas9 VP64 with ZFs or TALEs (blue). qRT-PCR showing MASPIN expression in HEK293T cells after transfection with a combination of ZF-97 and/or-126 VP64 **C.** or TALE-99 and/or -128 VP64 **D.** and dCas9 No Effector (CRISPR no-Eff) or dCas9 VP64 (CRISPR VP64) with or without MASPIN sgRNAs (sgRNA mix) as indicated in the figure. All qRT-PCR data indicate the fold change in MASPIN transcription levels relative to cells transfected with empty vector, normalized against GAPDH expression (error bars represent ± SEM, n=3). Statistical significance: one-way ANOVA, corrected for multiple comparisons using the Tukey HSD test, $p<0.0001$ (***), $p>0.05$ means differences were non-significant.

Supplementary Table S1: Binding sequences of each ATF for MASPIN and REPRIMO promoters

A

Artificial Transcription Factor Binding domain:	Binding sequence (5' – 3')	Strand
**Zinc Finger Protein (ZFP):**		
ZF -97	**GCAGAAGCAGCGGTGGCT**	-
ZF -126	**GCAGTGGGCGTGGCGGTG**	+
**Transcription Activator-Like Effector (TALE):**		
TALE -99	**TGAGCCACCGCTGCTTCTGC**	+
TALE -128	**TGGCAGTGGGCGTGGCGGTG**	+
**Single guide RNA (sgRNA) for CRISPR/dCas9 systems:**		
sgRNA -76	**GATGTGGAGGCGACCGTGTC <u>TGG</u>**	-
sgRNA -180	**GTAGGAGAGGAGTGCCGCCG <u>AGG</u>**	+
sgRNA -279	**GGCCTCCAACATGTTCGGGC <u>AGG</u>**	-
sgRNA -344	**GCAATCCTCTCGGCCCACGC <u>AGG</u>**	-

B

Artificial Transcription Factor Binding domain:	Binding sequence (5' – 3')	Strand
**Transcription Activator-Like Effector (TALE):**		
TALE -222	**TGCTCAGCGCAGCCACCCCG**	+
TALE -700	**TCCAACGCAGGCGAGTCGGA**	+
TALE -740	**TGTAAGAAACATTCTCAGAT**	+
**Single guide RNA (sgRNA) for CRISPR/dCas9 systems:**		
sgRNA -236	**GCCGGGAACGAGCACCACCA <u>GGG</u>**	-
sgRNA -305	**CGGCCCGCACCTCCCACGGC <u>CGG</u>**	-
sgRNA -372	**GCGCGGCGGTGCGGCCGGAA <u>GGG</u>**	+
sgRNA -618	**GCCACTTTCCATTCGCCCAG <u>AGG</u>**	-

All sequences are shown in 5′-3′ direction, and whether the ATF binds to the Plus (+) or Minus (-) strand is indicated For sgRNAs, the PAM is shown underlined.
